# Reversibility of Male Factor Infertility: Mechanisms and Evidence from the Literature

**DOI:** 10.3390/ijms27208968

**Published:** 2026-10-09

**Authors:** Sandro La Vignera, Aldo E. Calogero, Rossella Cannarella, Andrea Crafa, Federica Barbagallo, Rosita A. Condorelli

**Affiliations:** Department of Clinical and Experimental Medicine, University of Catania, 95123 Catania, Italy; acaloger@unict.it (A.E.C.); rossella.cannarella@unict.it (R.C.); crafa.andrea@outlook.it (A.C.); federica.barbagallo11@gmail.com (F.B.); rosita.condorelli@unict.it (R.A.C.)

**Keywords:** azoospermia, male infertility, normogonadotropic normotestosteronemic oligozoospermia, reversibility, spermiotoxic drugs, varicocelectomy

## Abstract

Male factor infertility contributes to approximately 50% of reproductive failure in couples worldwide. We conducted a narrative critical review synthesizing evidence from systematic reviews, meta-analyses, randomized controlled trials, and clinical practice guidelines on reversible and irreversible causes of male infertility, examining hormonal, surgical, pharmacological, lifestyle, and drug-induced etiologies. The majority of identifiable etiologies demonstrate some degree of reversibility. Varicocele (prevalence 25–40% in infertile men) achieves natural pregnancy rates of 30–40% after varicocelectomy. Hypogonadotropic hypogonadism responds to gonadotropin therapy with spermatogenesis induction in 90–95% of cases. Obstructive azoospermia achieves ductal patency rates of 85–97% with microsurgical reconstruction. Male accessory gland inflammation is reversible with prompt antibiotic therapy. Spermiotoxic drug exposures—including anabolic-androgenic steroids, sulfasalazine, and chemotherapy—show variable reversibility depending on agent and duration of exposure. Normogonadotropic normotestosteronemic oligozoospermia represents a commonly encountered phenotype that may respond to empirical medical therapy. Lifestyle modifications demonstrate measurable benefits in selected patients. Irreversible causes include Klinefelter syndrome, Y-chromosome AZFa/b microdeletions, and severe post-chemotherapy damage. Systematic andrological evaluation, including comprehensive drug history, is essential to identify treatable causes and guide clinical management.

## 1. Introduction

Male factor infertility represents a substantial contributor to reproductive dysfunction worldwide, affecting approximately 50% of infertile couples and serving as the sole cause in 20–30% of cases [1]. The condition encompasses a heterogeneous spectrum of disorders affecting sperm production, maturation, or transport, with etiologies ranging from genetic abnormalities and hormonal imbalances to anatomical defects, infections, drug exposures, and lifestyle factors [2]. Despite advances in assisted reproductive technology (ART), identifying and treating reversible causes of male infertility remains a clinical priority, as spontaneous conception avoids the costs, risks, and psychological burden associated with ART procedures [3].

The prevalence of male infertility varies globally, influenced by geographic, environmental, and socioeconomic factors [4]. Common etiologies include varicocele (present in 15–40% of infertile men), hormonal disorders, genetic abnormalities, infections, obstructive lesions, cryptorchidism, and iatrogenic or drug-induced causes [5,6]. Recent comprehensive guidelines from the Japanese Urological Association and other international bodies emphasize the multifactorial nature of male infertility and the importance of systematic evaluation to identify reversible causes [2,7].

Understanding the mechanisms underlying reversibility is essential for clinical decision-making. Reversibility depends on whether the primary lesion affects the hypothalamic–pituitary–gonadal (HPG) axis, testicular parenchyma, or excurrent ductal system, and whether the insult is functional (potentially reversible) or structural/genetic (often irreversible) [8]. Timely intervention is critical, as prolonged exposure to gonadotoxic factors or delayed treatment of anatomical abnormalities may result in irreversible testicular damage [9].

This comprehensive review synthesizes current evidence on the reversibility of male factor infertility, examining hormonal, surgical, pharmacological, lifestyle, and drug-induced etiologies. We provide a clinical decision algorithm to guide systematic evaluation and management, with particular emphasis on newly recognized reversible causes including spermiotoxic medications and male accessory gland inflammation (MAGI). Our objectives are to: (1) characterize the prevalence and mechanisms of reversible versus irreversible causes; (2) evaluate the evidence for specific interventions; (3) identify predictive factors for successful reversal; and (4) highlight critical knowledge gaps requiring future research.

For the purposes of this review, three levels of reversal are distinguished: (1) etiological reversal—elimination of the underlying cause (e.g., cessation of a spermiotoxic drug, surgical correction of varicocele or obstruction); (2) restoration of spermatogenesis or sperm transport—objective improvement in semen parameters sufficient to permit fertilization; and (3) achievement of natural pregnancy or live birth—the clinically most meaningful endpoint. Assisted reproductive technology (ART) with retrieved sperm represents circumvention rather than reversal of the underlying cause and is therefore classified separately. These distinctions are maintained throughout this review, and readers are cautioned that published studies frequently report different endpoints (semen biomarkers, sperm in ejaculate, pregnancy rate, live birth rate), which limits direct comparison.

## 2. Material and Methods

We included systematic reviews, meta-analyses, randomized controlled trials, prospective cohort studies, clinical practice guidelines, and high-quality retrospective studies published in English. We prioritized studies reporting quantitative outcomes including semen parameter improvements, pregnancy rates, and recovery timeframes. We excluded case reports (except for rare conditions), animal-only studies, and studies with insufficient methodological detail.

## 3. Results

### 3.1. Overview of Reversible and Irreversible Causes

**Figure 1:**
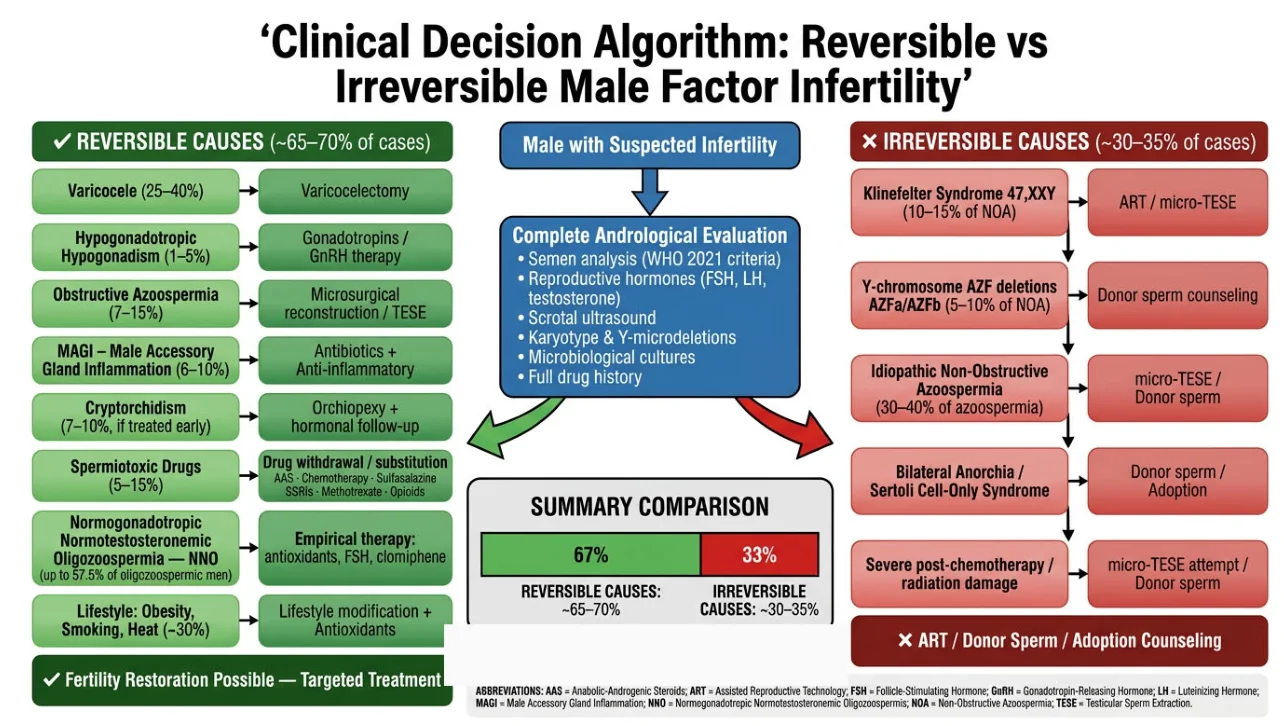
Clinical Decision Algorithm for Reversible versus Irreversible Male Factor Infertility. The flowchart depicts the initial andrological work-up (semen analysis, hormonal assessment, genetic testing, scrotal ultrasound, microbiological cultures, and systematic drug history) followed by categorization into two major pathways. Reversible causes (**left pathway**) include: normogonadotropic normotestosteronemic oligozoospermia (NNO; up to 57.5% of oligozoospermic infertile men); [10,11] varicocele (25–40%); hypogonadotropic hypogonadism (1–5%); obstructive azoospermia (7–15%); male accessory gland inflammation (MAGI; 6–10%); cryptorchidism (7–10%); spermiotoxic drugs (5–15%); and lifestyle factors (~30%). Irreversible causes (**right pathway**) include Klinefelter syndrome, Y-chromosome AZFa/b microdeletions (AZFc deletions carry more variable prognosis—sperm retrieval may be possible in some cases), idiopathic non-obstructive azoospermia, bilateral anorchia, Sertoli cell-only syndrome, and severe post-chemotherapy/radiation damage. Note: proportions depicted represent estimates from selected cohort data and should not be interpreted as fixed population parameters. AAS = anabolic-androgenic steroids; ART = assisted reproductive technology; FSH = follicle-stimulating hormone; LH = luteinizing hormone; MAGI = male accessory gland inflammation; micro-TESE = microdissection testicular sperm extraction; NNO = normogonadotropic normotestosteronemic oligozoospermia; NOA = non-obstructive azoospermia; SSRIs = selective serotonin reuptake inhibitors.

Male infertility etiologies can be classified as reversible, partially reversible, or irreversible based on the potential for spontaneous fertility restoration through medical, surgical, or lifestyle interventions (Figure 1). Table 1 summarizes the prevalence, mechanisms, and reversibility potential of major causes identified in large andrological cohorts. Table 2 details the principal spermiotoxic drug classes, their mechanisms of action, reversibility profiles, and management strategies. Table 3 presents the key prognostic factors associated with treatment outcomes across the major etiologies.

Reversible causes collectively account for approximately 50–70% of identifiable male infertility cases and include varicocele, hypogonadotropic hypogonadism, obstructive azoospermia, male accessory gland inflammation, spermiotoxic drug exposures, cryptorchidism (if treated early), lifestyle factors, and idiopathic oligozoospermia. Irreversible causes include Klinefelter syndrome (47,XXY), Y-chromosome AZF deletions, idiopathic non-obstructive azoospermia, bilateral anorchia or Sertoli cell-only syndrome, and severe post-chemotherapy or post-radiation testicular damage [5,31]. It should be noted that these categories are not mutually exclusive, frequently co-occur, and the overall percentage is not obtained by simple addition; furthermore, this estimate is derived from specialist andrology clinic cohorts and may overestimate the true population-level reversibility rate [27,32].

### 3.2. Varicocele

Varicocele, defined as abnormal dilatation of the pampiniform plexus veins, is the most common identifiable and surgically correctable cause of male infertility, with prevalence ranging from 25% to 40% in infertile men compared to 15% in the general male population. Large tertiary center cohorts report varicocele prevalence of 37.3% among infertile men [5,33,34].

#### 3.2.1. Pathophysiology

The mechanisms of varicocele-induced infertility include testicular hyperthermia, venous stasis, reflux of adrenal and renal metabolites, oxidative stress, and impaired Leydig and Sertoli cell function. These factors collectively damage germ cells and disrupt spermatogenesis, leading to progressive deterioration of semen parameters over time [35]. At the molecular level, testicular hypoxia stabilises HIF-1α, activating downstream gene-expression changes that impair germ-cell survival; heat-shock protein responses (HSP70/HSP90) and mitochondrial dysfunction further compromise spermatogenesis; ROS-mediated lipid peroxidation damages sperm plasma membranes; and impaired Leydig cell steroidogenesis occurs via reduced StAR protein expression, further lowering intratesticular testosterone [36].

#### 3.2.2. Reversibility Evidence

Varicocelectomy demonstrates significant improvements in semen parameters and pregnancy outcomes. A comprehensive meta-analysis of 4473 patients showed that varicocele repair significantly improved sperm concentration (mean difference +9.71 × 10^6^/mL), total motility (+9.69%), and progressive motility (+9.48%). Natural pregnancy rates after varicocelectomy range from 30% to 40% in most series [12]. A recent systematic review reported that varicocelectomy reduced sperm DNA fragmentation by a mean of 5.89% [13]. However, cross-study comparisons are limited by the use of non-interchangeable assays (SCSA, TUNEL, Comet, SCD) with different threshold criteria; furthermore, a causal link between post-varicocelectomy reductions in sperm DNA fragmentation and improvement in live-birth rates remains to be confirmed in prospective trials [25].

For men with non-obstructive azoospermia and clinical varicocele, varicocelectomy may induce sperm appearance in the ejaculate in carefully selected cases, though success rates are lower (approximately 20–30%) and patient selection criteria remain debated. Predictive factors for successful reversal include younger age, lower baseline FSH, larger testicular volume, and higher-grade varicocele [26,37,38].

#### 3.2.3. Clinical Management

Current guidelines recommend varicocelectomy for men with clinical varicocele, abnormal semen parameters, and otherwise unexplained infertility, particularly when the female partner has normal fertility potential [2,7]. Microsurgical subinguinal or inguinal approaches are preferred due to lower complication rates compared to laparoscopic or high ligation techniques [28].

### 3.3. Hypogonadotropic Hypogonadism

Hypogonadotropic hypogonadism (HH), characterized by low serum testosterone with low or inappropriately normal gonadotropins, represents a highly reversible cause of male infertility when due to functional suppression of the HPG axis rather than structural pituitary lesions [39]. The prevalence of HH among infertile men ranges from 1% to 5% in most series, though overall hypogonadism (including primary forms) may affect 10–37% depending on diagnostic criteria and population characteristics [5,29,40].

#### 3.3.1. Pathophysiology

HH results from reduced hypothalamic GnRH secretion or impaired pituitary gonadotropin (LH and FSH) production, leading to decreased intratesticular testosterone and impaired spermatogenesis. Causes include congenital conditions (Kallmann syndrome, idiopathic HH), acquired pituitary lesions, hyperprolactinemia, exogenous testosterone or anabolic steroid use, opioid medications, and obesity-related functional suppression [41,42,43].

#### 3.3.2. Reversibility Evidence

Gonadotropin therapy is highly effective for inducing spermatogenesis in men with HH. Treatment with human chorionic gonadotropin (hCG) alone or combined hCG plus recombinant FSH (rFSH) achieves sperm appearance in the ejaculate in 90–95% of men with congenital HH. A systematic review of 1868 men with HH treated with gonadotropins [10,11] reported median time to sperm appearance of 6–12 months, with 70–80% achieving sperm concentrations sufficient for natural conception or intrauterine insemination [44].

For men with iatrogenic HH due to exogenous testosterone or anabolic-androgenic steroid (AAS) use, cessation of the offending agent combined with selective estrogen receptor modulators (SERMs) such as clomiphene citrate or gonadotropin therapy can restore spermatogenesis, though recovery is variable and may take many months (see Section 3.7.1) [45,46].

#### 3.3.3. Clinical Management

Initial evaluation includes measurement of serum testosterone, LH, FSH, and prolactin, with pituitary MRI if a structural lesion is suspected [2]. Treatment options include hCG monotherapy (1500–3000 IU subcutaneously 2–3 times weekly) or combined hCG plus rFSH (75–150 IU subcutaneously 2–3 times weekly). SERMs (clomiphene citrate 25–50 mg daily) may be used for mild cases or as adjunctive therapy [47]. Treatment duration typically ranges from 6 to 24 months, with serial semen analyses every 3 months to monitor response [48,49].

### 3.4. Obstructive Azoospermia

Obstructive azoospermia (OA), defined as absence of sperm in the ejaculate due to bilateral obstruction of the excurrent ductal system despite normal spermatogenesis, accounts for 7–15% of azoospermic men and approximately 3–5% of all infertile men. OA is a highly reversible cause when anatomically amenable to surgical reconstruction [5,50].

#### 3.4.1. Pathophysiology

Obstruction may occur at the level of the epididymis, vas deferens, or ejaculatory ducts. Etiologies include congenital bilateral absence of the vas deferens (CBAVD, often associated with CFTR gene mutations), post-infectious epididymal obstruction (following epididymitis or sexually transmitted infections), iatrogenic injury (post-hernia repair, post-vasectomy), ejaculatory duct obstruction, and idiopathic causes [51,52,53].

#### 3.4.2. Reversibility Evidence

Microsurgical reconstruction achieves high patency and pregnancy rates. Vasovasostomy (VV) for vasectomy reversal achieves patency rates of 85–97% and natural pregnancy rates of 30–75%, with outcomes inversely related to obstructive interval. A large series of 1469 men undergoing VV [14,15] reported patency rates of 97% when obstructive interval was <3 years, declining to 71% for intervals >15 years [54].

Vasoepididymostomy (VE) for epididymal obstruction achieves patency rates of 65–85% and pregnancy rates of 20–40%. Transurethral resection of ejaculatory ducts (TURED) for ejaculatory duct obstruction shows variable success, with sperm appearance in 50–75% and pregnancy rates of 20–30% [55,56].

For men with unreconstructable obstruction or failed reconstruction, testicular sperm extraction (TESE) combined with intracytoplasmic sperm injection (ICSI) provides an alternative pathway to biological paternity, with fertilization rates of 50–70% and live birth rates of 25–35% per cycle; these figures derive from specialist centre series and are not currently supported by a single citation in the reference list.

#### 3.4.3. Clinical Management

Diagnostic evaluation includes physical examination, semen analysis showing azoospermia with normal semen volume (>1.5 mL), normal testicular size, and hormonal profile (normal FSH and testosterone). Scrotal ultrasound may identify epididymal dilatation or absence of the vas deferens. Genetic testing for CFTR mutations is indicated when CBAVD is suspected [57,58,59].

Microsurgical reconstruction is the preferred first-line approach when technically feasible, as it offers the potential for multiple natural pregnancies without ongoing ART costs. Patient counseling should address success rates, obstructive interval effects, and alternative options including TESE-ICSI [60,61].

### 3.5. Male Accessory Gland Inflammation (MAGI)

Male accessory gland inflammation (MAGI) encompasses inflammatory and infectious conditions affecting the epididymis, prostate, and seminal vesicles, representing an important but often underrecognized reversible cause of male infertility. MAGI affects approximately 6–10% of infertile men presenting to andrology clinics [16,62].

#### 3.5.1. Pathophysiology

MAGI results from ascending bacterial or sexually transmitted infections, with common pathogens including Chlamydia trachomatis, Mycoplasma genitalium, Ureaplasma urealyticum, Escherichia coli, and other uropathogens. The inflammatory cascade leads to leukocytospermia (>1 × 10^6^ [63,64] white blood cells/mL semen), oxidative stress, sperm DNA fragmentation, impaired sperm function, and potential ductal obstruction or scarring [65,66].

Clinical manifestations include acute or chronic epididymitis, prostatitis, and seminal vesiculitis. Acute epididymitis, if untreated or inadequately treated, carries significant risk of permanent sequelae: approximately 10% of men develop azoospermia and 30% develop oligozoospermia due to epididymal scarring and obstruction [17,67].

#### 3.5.2. Diagnostic Criteria

WHO diagnostic criteria for MAGI include: (1) leukocytospermia (>1 × 10^6^ peroxidase-positive white blood cells/mL); (2) positive microbiological cultures from semen, urethral swab, or expressed prostatic secretions; (3) elevated inflammatory markers in seminal plasma; and (4) ultrasound findings of epididymal thickening, prostatic calcifications, or seminal vesicle dilatation. The attribution of these specific criteria to WHO should be verified against the current WHO laboratory manual edition, as diagnostic thresholds have been revised in recent editions [68,69].

#### 3.5.3. Reversibility Evidence

Prompt antibiotic therapy combined with anti-inflammatory treatment can restore fertility in the majority of cases when initiated early. Published data indicate that prompt antibiotic therapy combined with anti-inflammatory treatment can improve semen parameters and achieve natural pregnancy in 30–40% of cases when initiated early [16,17,18,19,70].

However, delayed treatment or recurrent infections increase the risk of irreversible damage. Bilateral acute epididymitis carries the highest risk of permanent azoospermia (10%) and oligozoospermia (30%) if not treated promptly [17].

#### 3.5.4. Clinical Management

Systematic evaluation of all infertile men should include assessment for MAGI through semen leukocyte analysis, microbiological cultures, and scrotal ultrasound when clinically indicated [2]. Treatment consists of: (1) pathogen-directed antibiotic therapy for 4–6 weeks; (2) anti-inflammatory agents (NSAIDs); (3) treatment of sexual partners for sexually transmitted infections; and (4) follow-up semen analysis 3 months post-treatment to assess response. Prevention strategies include prompt treatment of urogenital infections, safe sexual practices, and avoidance of urethral instrumentation when possible [18,19,70].

### 3.6. Normogonadotropic Normotestosteronemic Oligozoospermia (NNO)

Normogonadotropic normotestosteronemic oligozoospermia (NNO) represents a clinically distinct and potentially treatable subgroup of male infertility characterized by reduced sperm concentration (<15 × 10^6^/mL) in the presence of normal serum FSH, LH, and testosterone levels [10,11]. This phenotype distinguishes itself from classical hypogonadotropic or hypergonadotropic states and represents a substantial proportion of oligozoospermic infertile men presenting to andrology clinics. It should be noted that NNO is a pragmatic clinical phenotype defined by the absence of detectable hormonal abnormalities on standard immunoassay, rather than a validated nosological entity with established pathognomonic criteria; its boundaries with other forms of idiopathic oligozoospermia are not sharply defined.

#### 3.6.1. Epidemiological Frequency

NNO is remarkably common among oligozoospermic infertile men. In a retrospective series of 113 infertile men with sperm concentration <15 × 10^6^/mL, 65 patients (57.5%) presented with a normal hormonal profile (normal testosterone, FSH, LH, TSH, and prolactin) at initial evaluation. Earlier studies similarly documented that a substantial proportion of oligozoospermic men maintain normal gonadotropin and testosterone levels despite impaired spermatogenesis [71,72]. These findings indicate that NNO represents the majority phenotype among oligozoospermic infertile men in clinical practice, challenging the assumption that oligozoospermia invariably reflects detectable hormonal abnormalities [10,11].

#### 3.6.2. Pathophysiological Mechanisms

The mechanisms underlying NNO are incompletely understood but likely multifactorial. Proposed pathophysiological contributors include: (1) subtle dysregulation of the HPG axis not detectable by standard immunoassays, including altered gonadotropin pulsatility, bioactivity, or receptor sensitivity; (2) oxidative stress and reactive oxygen species (ROS) generation leading to sperm membrane lipid peroxidation, mitochondrial dysfunction, and impaired sperm motility and morphology; (3) elevated sperm DNA fragmentation resulting from oxidative damage, defective chromatin packaging, or impaired apoptotic mechanisms; (4) epigenetic alterations affecting spermatogenesis, including aberrant DNA methylation patterns and histone modifications; and (5) unrecognized genetic [11] variants or polymorphisms affecting spermatogenic efficiency without causing overt hormonal derangement. The normal hormonal milieu in NNO suggests that the primary defect lies at the testicular level—either in spermatogenic efficiency, sperm maturation, or post-testicular sperm quality—rather than in central endocrine drive.

#### 3.6.3. Treatment Options

Management of NNO is largely empirical, given the heterogeneous and often unidentified underlying causes. WHO 2025 recommendations on fertility care note that the overall certainty of evidence for antioxidant therapy in male infertility is low [73], and guideline-supported indications should be distinguished from empirical off-label use; FSH supplementation and clomiphene citrate in normogonadotropic men remain off-label and investigational. Evidence-based and commonly employed treatment strategies include [44]:

- Antioxidant Therapy: Oral antioxidant supplementation (e.g., vitamin E, vitamin C, coenzyme Q10, N-acetylcysteine, selenium, zinc, L-carnitine) aims to reduce oxidative stress and improve sperm parameters. Multiple randomized controlled trials and meta-analyses have demonstrated modest but significant improvements in sperm concentration, motility, and DNA integrity with antioxidant therapy in idiopathic oligozoospermia, with some studies reporting increased pregnancy rates.
- FSH Supplementation: Exogenous recombinant FSH (typically 75–150 IU subcutaneously three times weekly for 3–6 months) has been used empirically in normogonadotropic oligozoospermia to augment spermatogenesis. Although baseline FSH is normal, the rationale is that supraphysiological intratesticular FSH levels may overcome subtle spermatogenic defects.
- Clomiphene Citrate: This selective estrogen receptor modulator increases endogenous gonadotropin secretion by blocking negative feedback at the hypothalamus and pituitary. Clomiphene citrate (25–50 mg daily for 3–6 months) has been used off-label in normogonadotropic oligozoospermia, with some studies reporting improvements in sperm parameters and pregnancy rates.
- Lifestyle Modification: Weight loss in obese men, smoking cessation, alcohol moderation, avoidance of testicular heat exposure, and optimization of sleep and stress management can improve semen parameters and fertility outcomes in men with NNO.
- Treatment of Subclinical Conditions: Systematic evaluation for and treatment of subclinical varicocele, genital tract infections, and medication effects may identify reversible contributors in some NNO cases.

#### 3.6.4. Reversibility Evidence

NNO demonstrates reversibility potential in a variable proportion of cases, though the evidence base derives primarily from small prospective studies and retrospective series. Response to empirical antioxidant therapy, hormonal stimulation, or lifestyle modification has been documented in published cohorts, with improvements in sperm concentration, motility, morphology, and DNA integrity observed in a proportion of treated men [74]; however, robust randomized controlled trial data specific to NNO as a distinct phenotype are limited. Spontaneous pregnancy following treatment has been reported in published cohorts, with higher success rates generally observed in men with milder oligozoospermia and shorter duration of infertility [10,11].

#### 3.6.5. Clinical Implications

Recognition of NNO as a distinct clinical entity has important implications for counseling and management. Men with oligozoospermia and normal hormonal profiles should be informed that their condition is common, potentially treatable, and associated with reasonable prospects for fertility improvement with empirical therapy. A trial of medical therapy (antioxidants, hormonal stimulation, lifestyle modification) for 3–6 months is reasonable before considering ART, particularly in couples with female age <35 years and no severe female factor. Serial semen analyses at 3-month intervals are recommended to monitor treatment response, given the 74-day spermatogenic cycle.

### 3.7. Spermiotoxic Drugs as Reversible Causes of Male Infertility

Drug-induced male infertility represents an important and often reversible cause affecting an estimated 5–15% of infertile men. Systematic drug history taking is essential in the evaluation of all infertile men, as many commonly prescribed and recreational substances can impair spermatogenesis through diverse mechanisms including HPG axis suppression, direct germ cell toxicity, oxidative stress, and endocrine disruption. Table 2 summarizes the principal spermiotoxic drug classes, their mechanisms of action, reversibility profiles, management strategies, and evidence quality [75,76].

#### 3.7.1. Anabolic-Androgenic Steroids and Exogenous Testosterone

Anabolic-androgenic steroids (AAS) and exogenous testosterone are among the most common and clinically significant causes of drug-related male infertility, particularly in young men. AAS and exogenous testosterone suppress the HPG axis through negative feedback, resulting in decreased endogenous LH and FSH secretion, reduced intratesticular testosterone concentrations (to <5% of normal levels), and profound impairment of spermatogenesis. At the molecular level, the intratesticular testosterone deficit (>95% reduction) impairs Sertoli cell androgen receptor (AR) signalling, downregulates FSH-receptor-mediated spermatogonial expansion, and disrupts blood–testis barrier integrity, collectively arresting spermatogenesis at the primary spermatocyte stage [30,77,78,79].

A retrospective cohort study of 66 men presenting to a fertility clinic with prior testosterone use found 91% had azoospermia at initial presentation. The prevalence of AAS use among infertile men varies by population but may exceed 10% in some cohorts, particularly among athletes and bodybuilders [20,80].

Recovery of spermatogenesis after AAS cessation is variable and often prolonged. The same cohort study reported median time to sperm recovery of 6.8 months (range 3–24 months), with approximately 65% achieving sperm concentrations >15 × 10^6^/mL. However, a substantial minority (20–35%) experience incomplete recovery or persistent azoospermia despite prolonged follow-up [20,81,82].

Case series demonstrate that medical therapy with clomiphene citrate (25–50 mg daily) or combined hCG plus FSH can accelerate recovery in some patients. Immediate cessation of AAS/testosterone is the first step when fertility is desired. Medical recovery strategies include: (1) SERMs (clomiphene citrate 25–50 mg daily or tamoxifen 20 mg daily) to stimulate endogenous gonadotropin secretion; (2) hCG (1500–3000 IU subcutaneously 2–3 times weekly) [83,84] plus rFSH (75–150 IU subcutaneously 2–3 times weekly) for severe or prolonged suppression; and (3) serial monitoring of semen parameters and serum hormones every 3 months. Early referral for ART should be considered for couples with persistent severe oligozoospermia or azoospermia after 12–18 months of medical therapy [85,86,87].

#### 3.7.2. Chemotherapy and Cytotoxic Agents

Cytotoxic chemotherapy produces dose- and agent-dependent gonadotoxicity, with alkylating agents (cyclophosphamide, chlorambucil, procarbazine) causing the most severe and often permanent testicular damage. Non-alkylating regimens (e.g., ABVD for Hodgkin lymphoma) [23,24] carry substantially lower gonadotoxic risk, with spermatogenesis recovery observed in 50–80% of patients within 2–3 years. Fertility preservation through semen cryopreservation before treatment initiation is strongly recommended for all men of reproductive age [23,38].

#### 3.7.3. Sulfasalazine

Sulfasalazine, used in inflammatory bowel disease and rheumatoid arthritis, causes reversible oligozoospermia and asthenozoospermia through direct sperm toxicity of the sulfapyridine moiety. Semen quality typically normalizes within 2–3 months of drug discontinuation or substitution with mesalazine. Pregnancy rates after cessation are comparable to the general infertile population [21,22].

#### 3.7.4. Antidepressants and Psychotropic Medications

Selective serotonin reuptake inhibitors (SSRIs) impair sperm DNA integrity, motility, and morphology through direct effects on sperm function and oxidative stress mechanisms [88]. Mechanistically, SERT (SLC6A4) is expressed on human sperm membranes; SSRI-mediated SERT inhibition disrupts serotonin-modulated sperm capacitation via altered cAMP/Ca^2+^ signalling, while ROS accumulation promotes abnormal chromatin packaging and fragmentation. Sperm quality typically improves within 3 months of drug discontinuation or substitution with agents with lower spermatotoxic profiles (e.g., bupropion) [89]. Clinicians should discuss fertility implications with young men initiating long-term SSRI therapy [65].

#### 3.7.5. Methotrexate and Immunosuppressants

Methotrexate at immunosuppressive doses causes transient spermatotoxicity and genotoxicity, with semen quality returning to baseline within approximately 3 months of cessation [90]. Men should discontinue methotrexate at least 3 months before attempting conception [91].

#### 3.7.6. Opioids and Recreational Drugs

Chronic opioid use suppresses the HPG axis, leading to hypogonadism and impaired spermatogenesis [9]. Recovery is variable and often incomplete. Cannabis, cocaine, and alcohol impair spermatogenesis through HPG suppression, oxidative stress, and direct germ cell toxicity. Cessation and addiction support services are recommended [30].

### 3.8. Lifestyle Factors and Reversible Infertility

Modifiable lifestyle factors represent an increasingly recognized and reversible contributor to male infertility. Obesity, smoking, alcohol consumption, heat exposure, and sedentary behavior all negatively impact semen quality through mechanisms including HPG axis dysregulation, oxidative stress, and endocrine disruption.

Weight loss of ≥10% body weight in obese men significantly improves testosterone levels, sperm concentration, and motility. Smoking cessation improves sperm DNA integrity and reduces oxidative stress. Avoidance of scrotal heat exposure (saunas, hot baths, prolonged sitting, tight underwear) may improve sperm parameters in susceptible individuals [92,93,94,95]. L-carnitine supplementation demonstrates modest but statistically significant improvements in sperm parameters and pregnancy rates in men with idiopathic infertility [27,32].

### 3.9. Irreversible Causes of Male Infertility

Several etiologies of male infertility are currently considered irreversible, necessitating ART with sperm retrieval, donor sperm, or adoption.

Klinefelter syndrome (47,XXY), the most common chromosomal cause of male infertility (affecting 10–15% of men with non-obstructive azoospermia), is associated with primary testicular failure [25]. However, microdissection TESE (micro-TESE) retrieves sperm in 40–70% of cases, enabling ICSI-based conception [13,25,96,97,98,99].

Y-chromosome AZFa and AZFb microdeletions are associated with absent or severely impaired spermatogenesis, with essentially no prospect for sperm retrieval. AZFc deletions carry more variable prognosis, with sperm retrieval possible in some cases [3,26,100].

Idiopathic non-obstructive azoospermia (NOA), accounting for 30–40% of azoospermic men, has limited treatment options beyond micro-TESE (sperm retrieval in 30–50%) or donor sperm [101,102,103]. Bilateral anorchia represents an absolute indication for donor sperm or adoption, as no testicular tissue is available for retrieval [104]. Sertoli cell-only syndrome (SCOS) is not uniformly irreversible: microdissection TESE identifies focal spermatogenesis in a subset of affected men, enabling ICSI-based conception, though success rates are variable and generally low; patients should be counselled individually regarding retrieval prospects [27,28,29,37,40].

## 4. Discussion

This comprehensive review demonstrates that male infertility is frequently reversible, with identifiable and treatable causes accounting for approximately 50–70% of cases presenting to specialized andrology clinics. The recognition of NNO as a distinct phenotype affecting up to 57.5% of oligozoospermic men, combined with the established reversibility of varicocele, HH, obstructive azoospermia, MAGI, and drug-induced infertility, underscores the critical importance of systematic andrological evaluation before proceeding to ART. This figure should be interpreted as an upper-bound estimate derived from specialist clinic populations, and the true population-level reversibility rate is likely lower given ascertainment bias [10,11].

In evaluating the evidence on reversibility, it is important to distinguish between different outcome levels: (1) improvement in semen biomarkers (e.g., sperm concentration, motility), which is the most commonly reported endpoint; (2) appearance of sperm in the ejaculate (relevant for men with initial azoospermia); (3) natural pregnancy rate; and (4) live birth rate, which is the clinically most meaningful endpoint. Most studies in this review report semen biomarker improvements or pregnancy rates; live birth data are less commonly available. Furthermore, couple fertility outcomes depend critically on female partner factors, including age, ovarian reserve, and tubal patency, which are not captured in most male infertility intervention studies. Clinicians should therefore interpret reported pregnancy rates in the context of the couple, not the male partner alone, and temper statements about ‘restoring natural fertility’ accordingly.

A key finding of this review is the high prevalence and reversibility of drug-induced male infertility. AAS and exogenous testosterone use, affecting an estimated 5–15% of infertile men in some populations, causes severe suppression of spermatogenesis that is frequently reversible with appropriate medical management. Similarly, sulfasalazine-induced oligozoospermia reverses rapidly after drug substitution [77,83], and SSRI-related sperm dysfunction improves within months of cessation or substitution [88]. These findings emphasize that a systematic drug history is an essential component of the andrological evaluation.

The clinical decision algorithm presented in Figure 1 provides a practical framework for systematizing the evaluation of male infertility. By stratifying patients into reversible and irreversible categories based on comprehensive andrological work-up, clinicians can prioritize targeted interventions and avoid premature recourse to ART. This approach has important implications for patient counseling, healthcare costs, and psychological well-being.

Several limitations of this review should be acknowledged. First, the evidence base for some etiologies (particularly MAGI and NNO) relies on relatively small prospective studies and retrospective series, limiting the strength of conclusions. Second, the definition and diagnostic criteria for some conditions (e.g., MAGI, subclinical varicocele) remain contested, potentially affecting prevalence estimates. Third, long-term fertility outcomes following treatment of reversible causes are incompletely characterized in many studies. Fourth, the impact of combined etiologies on reversibility potential has not been systematically evaluated. Fifth, the majority of reversibility evidence derives from uncontrolled before–after series susceptible to regression-to-the-mean bias, which may inflate apparent treatment effects. Sixth, publication bias and small-study effects have not been formally assessed. Seventh, an English-language restriction was applied to the literature search, which was not previously declared as a limitation and may have led to the exclusion of relevant non-English data.

Future research priorities include: (1) prospective multicenter studies evaluating long-term fertility outcomes after treatment of reversible causes; (2) development and validation of predictive biomarkers for treatment response; (3) standardization of diagnostic criteria for MAGI and NNO; (4) investigation of combined treatment approaches for men with multiple contributing etiologies; and (5) health economic analyses comparing treatment of reversible causes versus direct ART.

## 5. Conclusions

Male infertility is frequently reversible through targeted interventions. Systematic andrological evaluation—encompassing semen analysis, reproductive hormones, genetic testing, scrotal ultrasound, microbiological assessment, and comprehensive drug history—is essential to identify treatable causes. Reversible etiologies including varicocele, hypogonadotropic hypogonadism, obstructive azoospermia, MAGI, normogonadotropic normotestosteronemic oligozoospermia, spermiotoxic drug exposures, and lifestyle factors collectively account for the majority of identifiable male infertility cases. Recognition and treatment of these conditions can improve fertility outcomes and, in appropriate cases, reduce reliance on ART; however, the most clinically meaningful endpoint is live birth, and couple-level factors—including female partner age and reproductive reserve—must be incorporated into clinical decision-making. Future research should focus on live-birth outcomes, predictive biomarkers for treatment response, and standardization of diagnostic criteria.

## Figures and Tables

**Table 1:** Summary of Reversible and Irreversible Causes of Male Infertility: Prevalence, Mechanisms, Reversibility, and Evidence Quality.

Etiology	Prevalence (%)	Primary Mechanism	Reversibility	Recovery Timeframe	Evidence Quality
Varicocele	25–40	Oxidative stress, hyperthermia	Yes (30–40% pregnancy rate) [12,13]	3–12 months	Moderate
Hypogonadotropic hypogonadism	1–5	HPG axis suppression	Yes (90–95% spermatogenesis induction) [10,11]	6–24 months	High
Obstructive azoospermia	7–15	Ductal obstruction	Yes (85–97% patency after VV) [14,15]	Immediate post-surgery	High
MAGI	6–10	Infection/inflammation	Yes (if treated promptly) [16,17,18,19]	3–6 months	Low–Moderate
NNO	Up to 57.5 of oligozoospermic men	Multifactorial (oxidative stress, subtle HPG dysregulation)	Partial (30–50% response) [10,11]	3–6 months	Low
AAS/testosterone use	5–15	HPG suppression	Variable (65% recover) [20]	6.8 months median	Low–Moderate
Sulfasalazine	~5	Direct sperm toxicity	Yes [21,22]	2–3 months	Moderate
Chemotherapy (alkylating)	Variable	Direct germ cell toxicity	Often irreversible [23,24]	Variable/permanent	Moderate
Obesity/lifestyle	~30	Hormonal disruption, oxidative stress	Partial	6–12 months	Low
Klinefelter syndrome	10–15 of NOA	Primary testicular failure	No (micro-TESE in 40–70%) [13,25]	N/A	Moderate
AZFa/b deletions	5–10 of NOA	Spermatogenic failure	No [26]	N/A	High
Idiopathic NOA	30–40 of azoospermia	Unknown	No (micro-TESE 30–50%) [27,28,29]	N/A	Low

AAS = anabolic-androgenic steroids; HPG = hypothalamic–pituitary–gonadal; MAGI = male accessory gland inflammation; NNO = normogonadotropic normotestosteronemic oligozoospermia; NOA = non-obstructive azoospermia; VV = vasovasostomy. Note: The ‘Reversibility’ column reflects heterogeneous endpoints across studies (semen parameter improvement, natural pregnancy, live birth); direct comparison between conditions should be made with caution. Prevalence estimates are derived from specialized andrology clinic cohorts and may not be representative of the general male population. Evidence Quality was assigned based on the highest available study design (High = RCTs/meta-analyses with consistent results; Moderate = smaller RCTs or consistent observational data; Low = case series, retrospective, or limited data).

**Table 2:** Spermiotoxic Drugs: Mechanisms, Reversibility, Management, and Evidence Quality.

Drug Class	Examples	Mechanism	Reversibility	Recovery Timeframe	Management	Evidence Quality
AAS/Testosterone	Testosterone enanthate, nandrolone, stanozolol	HPG suppression	Variable; 65% recover [20]	6.8 months median	Cessation; SERMs or hCG + FSH; semen cryopreservation	Low–Moderate
Alkylating chemotherapy	Cyclophosphamide, chlorambucil, procarbazine	Direct germ cell DNA damage	Often irreversible [23,24]	Variable/permanent	Fertility preservation before treatment; micro-TESE	Moderate
Non-alkylating chemotherapy	ABVD (doxorubicin, bleomycin, vinblastine, dacarbazine)	Indirect germ cell toxicity	Yes (50–80%) [23]	2–3 years	Fertility preservation; monitor semen annually	Moderate
Sulfasalazine	Sulfasalazine	Direct sperm toxicity (sulfapyridine moiety)	Yes [21,22]	2–3 months	Substitute with mesalazine; reassess semen 3 months post-switch	Moderate
SSRIs	Paroxetine, sertraline, fluoxetine	Sperm DNA damage, motility impairment	Yes	~3 months	Substitution with bupropion; discuss fertility implications before initiating	Low–Moderate
Methotrexate	Methotrexate	Transient spermatotoxicity, genotoxicity	Yes	~3 months	Discontinue 3 months before conception attempts	Low
Opioids	Tramadol, methadone, morphine	HPG suppression, direct testicular toxicity	Variable; often incomplete [9]	Variable	Opioid-sparing strategies; hormonal assessment	Low
Recreational drugs	Cannabis, cocaine, alcohol	HPG suppression, oxidative stress, DNA damage	Variable [30]	Variable	Cessation; addiction support services	Low

AAS = anabolic-androgenic steroids; HPG = hypothalamic–pituitary–gonadal; SERMs = selective estrogen receptor modulators; hCG = human chorionic gonadotropin; FSH = follicle-stimulating hormone; SSRIs = selective serotonin reuptake inhibitors. Note: Clinical management recommendations in this table represent clinical practice consensus and expert opinion. Bupropion substitution for SSRIs and SERMs/hCG/FSH for AAS recovery should be considered investigational and require individualized benefit-risk assessment; recommendations for specific agents and doses should be implemented under specialist guidance. Evidence Quality: High = RCTs/meta-analyses; Moderate = smaller RCTs or consistent observational data; Low = case series or limited data.

**Table 3:** Prognostic Factors Associated with Outcome by Etiology (based on observational data; not validated prediction models).

Etiology	Favorable Prognostic Factors	Unfavorable Prognostic Factors	Evidence Quality
Varicocelectomy	Younger age, higher-grade varicocele, bilateral varicocele, lower baseline FSH, larger testicular volume	Older age (>40 years), low-grade varicocele, elevated FSH (>10 IU/L), small testicular volume (<15 mL)	Low (observational/retrospective)
Hypogonadotropic hypogonadism	Congenital HH, shorter duration of hypogonadism, larger baseline testicular volume (>4 mL), lower baseline FSH	Acquired pituitary lesions, prolonged hypogonadism, very small testes (<2 mL), history of cryptorchidism	Low (observational/retrospective)
Obstructive azoospermia (vasectomy reversal)	Shorter obstructive interval (<3 years), presence of sperm in vasal fluid, larger testicular volume	Longer obstructive interval (>15 years), absence of sperm in vasal fluid, testicular atrophy, sperm antibodies	Low (observational/retrospective)
MAGI	Prompt treatment initiation, acute presentation, identification of pathogen, absence of ductal obstruction	Delayed treatment, chronic/recurrent infections, bilateral epididymal involvement, established ductal obstruction	Low (observational/retrospective)
AAS-induced azoospermia	Shorter duration of use (<2 years), lower cumulative dose, younger age, prompt medical therapy	Prolonged use (>5 years), high cumulative doses, older age, delayed presentation	Low (observational/retrospective)
Chemotherapy-induced azoospermia	Non-alkylating regimens, lower cumulative doses, younger age at treatment, normal pre-treatment semen	Alkylating agents (procarbazine), high cumulative doses, older age, abnormal pre-treatment semen	Low (observational/retrospective)
Obesity-related infertility	Greater weight loss (>10% body weight), younger age, shorter duration of obesity, absence of comorbidities	Minimal weight loss (<5%), older age, long-standing obesity, metabolic syndrome	Low (observational/retrospective)
Idiopathic oligozoospermia	Milder oligozoospermia (>5 × 10^6^/mL), normal FSH, absence of varicocele, younger age	Severe oligozoospermia (<1 × 10^6^/mL), elevated FSH, advanced paternal age (>45 years)	Low (observational/retrospective)

FSH = follicle-stimulating hormone; HH = hypogonadotropic hypogonadism; MAGI = male accessory gland inflammation; AAS = anabolic-androgenic steroids. All data observational. Note: All prognostic factors listed in this table are derived from observational cohort or retrospective series data; they have not been validated in prospective prediction models. Quantitative thresholds are approximate and should be interpreted with caution.

## Data Availability

No new data were created or analyzed in this study. Data sharing is not applicable to this article.
